# Sustainable Innovations in Food Microbiology: Fermentation, Biocontrol, and Functional Foods

**DOI:** 10.3390/foods14132320

**Published:** 2025-06-30

**Authors:** Amanda Priscila Silva Nascimento, Ana Novo Barros

**Affiliations:** 1 Unidade Académica Engenharia de Alimentos, Universidade Federal Campina Grande, Av. Aprigio Veloso, 882, Campina Grande 58429-900, PA, Brazil; amandapriscil@yahoo.com.br; 2 Centre for the Research and Technology of Agro-Environmental and Biological Sciences (CITAB), University of Trás-os-Montes e Alto Douro (UTAD), 5000-801 Vila Real, Portugal

**Keywords:** food microbiology, sustainable fermentation, protective cultures, microbial biocontrol, functional foods, probiotics and postbiotics, food bioeconomy, omics technologies

## Abstract

The growing demand for more sustainable food systems has driven the development of solutions based on food microbiology, capable of integrating safety, functionality, and environmental responsibility. This paper presents a critical and up-to-date review of the most relevant advances at the interface between microbiology, sustainability, and food innovation. The analysis is structured around three main axes: (i) microbial fermentation, with a focus on traditional practices and precision technologies aimed at valorizing agro-industrial waste and producing functional foods; (ii) microbial biocontrol, including the use of bacteriocins, protective cultures, bacteriophages, and CRISPR-Cas (Clustered Regularly Interspaced Short Palindromic Repeats–CRISPR-associated)-based tools as alternatives to synthetic preservatives; and (iii) the development of functional foods containing probiotics, prebiotics, synbiotics, and postbiotics, with the potential to modulate the gut microbiota and promote metabolic, immune, and cognitive health. In addition to reviewing the microbiological and technological mechanisms involved, the paper discusses international regulatory milestones, scalability challenges, and market trends related to consumer acceptance and clean labeling. Finally, emerging trends and research gaps are addressed, including the use of omics technologies, artificial intelligence, and unexplored microbial resources. Food microbiology, by incorporating sustainable practices and advanced technologies, is positioned as a strategic pillar for building a healthy, circular, science-based food model.

## 1. Introduction

The pursuit of more sustainable food systems has become a critical global priority in the 21st century. Current estimates attribute up to 34% of global greenhouse gas emissions to the agro-food sector [1], which simultaneously places significant pressure on natural resources, driving environmental degradation, biodiversity loss, and depletion of soil and water reserves. Moreover, roughly one-third of all food produced worldwide is lost or wasted, intensifying both environmental and economic burdens [2]. These alarming statistics underscore the urgent necessity for a transformative shift toward food systems that harmonize productivity, technological innovation, and environmental stewardship.

Within this context, food microbiology emerges as a pivotal discipline to advance sustainability across the food production continuum. The application of microorganisms in fermentation, biocontrol, and functional food development has yielded promising sustainable solutions characterized by lower energy consumption, minimized waste generation, and enhanced the valorization of agro-industrial by-products [3]. Microorganisms have been indispensable throughout human history in food processing and preservation, from traditional fermentation techniques to cutting-edge approaches involving genetic engineering and synthetic biology.

Microbial fermentation constitutes a foundational pillar in this transition. Beyond the production of widely consumed fermented foods such as yogurt, sauerkraut, kefir, miso, and tempeh, fermentation processes are increasingly employed to convert agro-industrial residues into value-added products, thereby contributing to circular bioeconomy models with reduced environmental footprints [4,5]. These processes not only promote food diversification and safety but also support sustainable resource management.

Similarly, microbial biocontrol offers a natural and effective strategy to address food preservation challenges and reduce reliance on synthetic additives. Microorganisms that produce antimicrobial agents—including bacteriocins, organic acids, and peroxides—have demonstrated strong efficacy against pathogens and spoilage organisms while maintaining food quality and safety [6,7]. Recent advances, such as protective cultures, bacteriophage applications, and CRISPR-Cas technologies, have further enhanced the precision and sustainability of biocontrol measures, lowering environmental and toxicological risks.

The development of microbial functional foods represents another transformative avenue driven by advances in understanding the gut microbiota’s impact on human health, encompassing immunity, metabolism, cognitive function, and chronic disease risk [7,8]. Functional food formulations containing probiotics, prebiotics, synbiotics, and postbiotics not only improve nutritional profiles but also resonate with consumer demand for natural, health-promoting, and personalized dietary options.

In light of these developments, this review critically examines the latest sustainable innovations at the intersection of food microbiology and food systems. The discussion is organized around three thematic axes: (i) microbial fermentation for sustainable food production, (ii) microbial biocontrol as an eco-friendly alternative to chemical preservatives, and (iii) functional foods designed to enhance health outcomes. By synthesizing the current scientific evidence and identifying knowledge gaps, this work aims to guide future research and contribute to the design of resilient, circular food systems aligned with the United Nations Sustainable Development Goals (SDGs).

Thus, this review aims to provide a critical and integrative overview of recent sustainable innovations in food microbiology, focusing on microbial fermentation, microbial biocontrol, and functional foods. By synthesizing the current scientific knowledge and identifying technological and regulatory gaps, we intend to highlight how microbiological strategies can support circular food systems, health promotion, and environmental responsibility, thereby contributing to the scientific foundations of the Sustainable Development Goals (SDGs).

## 2. Microbial Fermentation for Sustainable Food Production

### 2.1. Traditional Fermented Foods: Cultural Heritage and Microbial Ecology

Traditional fermented foods constitute one of the oldest and most culturally rich forms of food biotechnology, rooted in empirical knowledge accumulated over thousands of years. These fermentations not only serve to preserve food but also enhance its digestibility, nutritional profile, and functional properties, contributing to human health while ensuring microbiological safety. Examples such as yogurt, kefir, tempeh, sauerkraut, miso, kimchi, and dadiah illustrate diverse regional practices shaped by local sociocultural contexts and ecological conditions.

The fermentation processes in these foods are driven by complex microbial consortia, predominantly composed of lactic acid bacteria (LAB), yeasts, and filamentous fungi. The composition of these microbial communities varies significantly according to the substrate, environmental factors, indigenous microbiota, and traditional production methods [4].

In yogurt production, controlled fermentation involves a symbiotic relationship between *Streptococcus thermophilus* and *Lactobacillus delbrueckii* subsp. *bulgaricus*, which cooperatively produce lactic acid to induce protein coagulation and develop volatile compounds responsible for its characteristic flavor and texture. Kefir grains form a natural symbiotic matrix of proteins and polysaccharides that harbor a diverse microbial consortium including LAB, yeasts such as *Kluyveromyces marxianus*, and acetic acid bacteria [9].

*Tempeh*, a traditional Indonesian fermented food, relies on the filamentous fungus *Rhizopus oligosporus*, which hydrolyzes soy proteins and lipids, releasing peptides with antioxidant, hypocholesterolemic, and antimicrobial activities [10]. Miso, a fermented soybean paste often combined with rice or barley, undergoes a multi-step fermentation starting with *Aspergillus oryzae* in the *koji* stage (i.e., the initial solid-state incubation step where *Aspergillus oryzae* grows on substrates such as soybeans or rice to generate a high-enzyme biomass inoculum), followed by fermentation by osmophilic yeasts (*Zygosaccharomyces rouxii*) and LAB, resulting in a product rich in free amino acids, vitamins, and aromatic compounds [11]. A summary of representative traditional fermented foods, their predominant microorganisms, fermentation methods, origin, and main metabolic products is provided in Table 1, which complements the microbiological and functional descriptions detailed in this section.

*Sauerkraut* exemplifies spontaneous vegetable fermentation, where species such as *Leuconostoc mesenteroides*, *Lactiplantibacillus plantarum*, and *Limosilactobacillus fermentum* drive acidification, inhibit spoilage and pathogenic microbes, and stabilize the food matrix. This fermentation is characterized by natural microbial successions influenced by pH gradients and substrate availability [12].

Recent research by Thierry et al. (2023) [13] underscored the microbial diversity and ecological resilience in traditional fermentation systems. Their analysis of 75 homemade vegetable fermentation samples from France revealed over 20 dominant species well-adapted to acidic environments, including tolerance to low pH and the capacity to synthesize volatile metabolites. These dynamics are governed by fundamental ecological mechanisms such as selection, dispersal, genetic drift, and speciation, rendering fermented foods valuable model systems for applied microbial ecology studies [14].

Beyond their cultural and historical significance, traditional fermented foods deliver documented nutritional and functional benefits. These include enhanced mineral bioavailability, degradation of antinutritional factors, biosynthesis of B vitamins, production of bioactive compounds, and beneficial modulation of the gut microbiota [15,16]. Preserving and investigating these time-honored practices offers an effective strategy to promote sustainable food systems, leveraging microbial biodiversity and low-cost biotechnological innovation.

### 2.2. Controlled and Precision Fermentation

Controlled and precision fermentation represent important advances in modern food biotechnology, building upon the foundations of traditional fermentation by incorporating contemporary microbiological methods, genetic engineering, and synthetic biology. These innovations aim to optimize fermentation processes, ensuring greater consistency, safety, and reproducibility of the final products [17,18].

The use of starter cultures allows for targeted control over microbial growth by inoculating selected strains with desirable traits, such as rapid acidification, production of flavor compounds, vitamin biosynthesis, or the ability to inhibit spoilage and pathogenic microorganisms [17]. Furthermore, multi-species microbial consortia that engage in synergistic metabolic interactions have shown promising results in generating fermented foods with enhanced sensory profiles and improved ecological stability in both artisanal and industrial contexts [18,19].

Recent developments in genetic engineering and synthetic biology have made it possible to tailor microorganisms to produce specific metabolites efficiently. These include essential amino acids, vitamins, enzymes, organic acids, and functional proteins. A notable example is the genetic modification of yeasts for the production of recombinant casein, which enables the creation of animal-free cheeses with a substantially lower environmental footprint [17].

Advanced genome-editing technologies such as CRISPR-Cas are widely applied to insert, delete, or modulate genes relevant to fermentation processes, while synthetic genetic circuits provide dynamic regulation of microbial metabolism. These tools have significantly improved both the biochemical efficiency and scalability of fermentation at the industrial level [20].

Precision fermentation has also paved the way for innovative animal-free alternatives, including milk, eggs, and meat substitutes. Companies like Perfect Day, Formo, and The EVERY Company utilize genetically engineered microorganisms—such as yeasts and filamentous fungi—to produce proteins like beta-lactoglobulin and albumin. These proteins replicate the techno-functional and sensory qualities of their animal-derived equivalents, meeting the growing consumer demand for ethical, sustainable, and allergen- or intolerance-friendly food products [21].

Moreover, the integration of artificial intelligence (AI) with precision fermentation is enhancing the development of novel foods. For instance, the Chilean startup NotCo employs an AI platform named “Giuseppe” that matches plant-based ingredients with molecular signatures of animal-based products. By combining extensive molecular databases, machine learning algorithms, and fermentation biotechnology, NotCo creates plant-based foods that closely replicate the flavor, texture, and performance of traditional animal products. This synergy between biotechnology and data science is opening new avenues for innovation in sustainable food production [22].

Furthermore, the global market for precision fermentation ingredients was valued at approximately USD 2.8 billion in 2023 and is projected to reach over USD 36.3 billion by 2030, with a compound annual growth rate (CAGR) of around 44% [23]. This trend underscores the rapid industrial expansion and strong investment interest in this emerging field.

Despite the promise of these microbial technologies, their large-scale implementation in low- and middle-income countries (LMICs) faces significant challenges related to infrastructure, regulatory capacity, and investment. The high initial cost of bioreactors, need for specialized personnel, and limitations in cold-chain logistics can hinder scalability. Moreover, affordable access to microbial cultures and proprietary biotechnological tools remain restricted in many regions. Although comprehensive global data are still lacking, addressing these barriers requires public–private partnerships, technology transfer, and regionally adapted strategies to ensure equitable innovation in food systems [24].

However, the implementation of such innovative microbial technologies is not without potential drawbacks. Concerns have been raised regarding the unintended ecological consequences, such as the loss of native microbial diversity and the production of unexpected or harmful metabolites in complex food ecosystems. In particular, the use of genetically modified (GM) starter cultures, while offering benefits such as improved performance and safety, remains controversial. These concerns are often rooted in ethical debates, environmental risk perceptions, and consumer resistance, especially in regions where regulatory and labeling frameworks for GMOs are still evolving [25].

### 2.3. Valorization of Agro-Industrial Waste Through Fermentation

The growing generation of agro-industrial waste represents one of the main challenges for the sustainability of food systems. It is estimated that globally, around 1.3 billion tons of food are wasted annually, resulting in economic losses of approximately USD 940 billion and significant environmental impacts, such as greenhouse gas emissions, inefficient water use, and soil contamination [2]. In this context, microbial fermentation emerges as a promising biotechnological strategy for the valorization of these wastes, allowing their conversion into bioactive compounds, functional ingredients, and high-value biomaterials.

Among the most efficient approaches is solid-state fermentation (SSF), which uses low-moisture agro-industrial substrates as support for microbial growth. Studies show that SSF enables the production of industrial enzymes, such as cellulases, pectinases, and xylanases, from substrates like sugarcane bagasse, rice husks, and fruit residues, with potential applications in the food, textile, and pharmaceutical sectors [26]. Additionally, SSF has been employed in the production of bioactive compounds, including phenolic antioxidants and organic acids, with preservative and nutraceutical functions [27,28].

Quantitative studies report that SSF can convert 30–80% of lignocellulosic biomass into value-added compounds such as organic acids, enzymes, and phenolic extracts, depending on the substrate and microbial consortium used [24]. For example, the global availability of agro-industrial waste suitable for SSF-based valorization exceeds 5 billion tons annually, especially from sugarcane, wheat, rice, and fruit processing [25]. Yields from SSF processes include ~12 g/L of citric acid from sugarcane bagasse using *Aspergillus niger* and protease activities above 900 U/g of dry substrate from soybean meal fermentation. These values reinforce the industrial viability of SSF as an eco-efficient biotechnological platform.

The fermentation of agro-industrial waste has also been applied to the production of biopolymers and biofuels. Lignocellulosic wastes, such as corn straw, cassava peels, and sugarcane bagasse, can be used as substrates for the synthesis of polyhydroxybutyrate (PHB) by microorganisms like *Bacillus megaterium* or *Cupriavidus necator*, contributing to the replacement of conventional plastics with biodegradable [2]. Furthermore, carbohydrate-rich wastes, such as molasses, whey, and tropical fruit residues, have been successfully used in bioethanol production, promoting the diversification of the energy matrix and reducing dependence on fossil fuels [29].

In the food industry, the fermentation of waste has been explored for the development of functional and nutritionally enriched ingredients. The production of single-cell proteins (SCPs) from nutrient-rich waste, such as fruit peels, vegetable scraps, and by-products from the brewing industry, has been proposed as a sustainable alternative for obtaining proteins for human and animal nutrition [30].

In parallel, fermentation processes have been applied in the extraction or synthesis of prebiotic compounds, antioxidant polyphenols, exopolysaccharides, and bioactive peptides, which can be incorporated into food formulations with functional and health claims [31].

Thus, the valorization of agro-industrial waste through fermentation not only reduces the environmental impact of the agro-industry but also promotes circular bioeconomy, contributing to sustainable innovation in food, energy, and biomaterials.

## 3. Microbial Biocontrol for Food Safety and Preservation

### 3.1. Natural Antimicrobials Produced by Microorganisms

Increasing awareness of the potential health risks and environmental impacts associated with synthetic chemical preservatives has driven the demand for natural and sustainable alternatives in food preservation. In this context, natural antimicrobials produced by microorganisms, including bacteriocins, organic acids, hydrogen peroxide, and certain phenolic derivatives, have gained significant attention as effective tools for microbial control, particularly in minimally processed foods [6].

Bacteriocins represent the most extensively studied group of microbial-derived antimicrobial peptides. These ribosomally synthesized compounds, predominantly produced by lactic acid bacteria (LAB), are capable of inhibiting spoilage organisms and foodborne pathogens such as *Listeria monocytogenes*, *Staphylococcus aureus*, and *Clostridium botulinum*. A prominent example is nisin, synthesized by *Lactococcus lactis*, which has been approved for use in a variety of food products, including cheeses, processed meats, and canned goods, due to its broad antimicrobial spectrum and established safety profile [6].

In addition to bacteriocins, LAB contributes to food preservation through the production of organic acids—notably lactic, acetic, and propionic acids—which reduce the pH of the surrounding environment, thereby inhibiting the growth of undesirable microorganisms. These acids act synergistically with other LAB-derived metabolites such as hydrogen peroxide and diacetyl, exerting both bacteriostatic and bactericidal effects, especially against Gram-negative bacteria [32].

Furthermore, some microorganisms are capable of biotransforming plant-derived phenolic compounds into more potent antioxidants and antimicrobial derivatives. This metabolic capability extends the functional potential of microbial fermentation beyond preservation, offering added value in the development of functional foods. For instance, strains of *Lactiplantibacillus plantarum* and *Pediococcus acidilactici* have been shown to produce antimicrobial compounds during the fermentation of plant-based substrates such as fruit juices, peels, and pulps [7,32].

The application of natural microbial antimicrobials is particularly advantageous in minimally processed foods, including ready-to-eat vegetables, refrigerated juices, artisanal cheeses, cured meats, and fermented products. In these categories, where thermal treatments or synthetic additives are often limited due to sensory, nutritional, or regulatory concerns, microbial-derived compounds provide microbiological safety while supporting clean label formulations aligned with consumer preferences [33].

Nonetheless, several challenges remain for the broader industrial application of these natural antimicrobials. Limitations such as thermal instability, reduced efficacy across diverse food matrices, and narrow spectra of antimicrobial activity must be addressed. To overcome these barriers, strategies including microencapsulation, synergistic use with other preservation hurdles, and co-application with protective microbial cultures are being explored to enhance stability, efficacy, and scalability [34,35,36].

In conclusion, antimicrobials produced by microorganisms offer a promising avenue for the development of safe, natural, and eco-conscious food preservation systems. Their integration into modern food processing supports both the principles of barrier technology and the transition toward cleaner, more sustainable production models within the food industry [37,38].

### 3.2. Competitive Exclusion and Protective Cultures

Competitive exclusion is a fundamental ecological principle in food microbiology, asserting that two microorganisms occupying similar ecological niches cannot stably coexist in the same environment, as one will eventually outcompete the other [32]. This concept underpins the development of protective cultures, a consortia of selected microorganisms that rapidly colonize the food matrix and inhibit the proliferation of pathogens and spoilage organisms through non-antibiotic mechanisms.

The effectiveness of competitive exclusion in food preservation is attributed to several microbial strategies, including the production of antimicrobial metabolites (e.g., bacteriocins and organic acids), competition for nutrients, acidification of the environment, and the release of hydrogen peroxide, siderophores, and volatile inhibitory compounds. Additionally, physical occupation of ecological niches limits the ability of undesirable microorganisms to establish themselves [32,33,34,35,36,37,38,39]. These mechanisms are particularly relevant in minimally processed foods and under refrigerated storage, where psychotropic pathogens such as Listeria monocytogenes pose significant food safety risks.

Protective cultures are commonly composed of lactic acid bacteria (LAB), including *Lactiplantibacillus plantarum*, *Lacticaseibacillus rhamnosus*, *Pediococcus acidilactici*, and *Leuconostoc mesenteroides*. These strains are selected for their adaptability to diverse substrates and resilience under variable conditions of pH, temperature, and water activity [40]. The use of autochthonous strains, isolated from the same food matrix, is encouraged to enhance colonization efficiency and preserve the traditional sensory attributes of the final product [41,42,43,44].

Applications of protective cultures are particularly well established in meat products, fresh cheeses, and fermented sausages. For instance, the incorporation of *Lactobacillus sakei* or *Carnobacterium maltaromaticum* in white cheeses and refrigerated dairy products has been shown to effectively control *Listeria monocytogenes* without negatively affecting sensory quality [40]. In minimally processed vegetables, microbial consortia comprising *Lactiplantibacillus* and *Pediococcus* spp. have demonstrated efficacy against Salmonella and Escherichia coli through acidification and competitive metabolic interactions [44].

The use of protective cultures aligns with clean label initiatives, being recognized as safe and natural preservation strategies. In many jurisdictions, LAB used in foods are designated as GRAS (Generally Recognized As Safe) or hold QPS (Qualified Presumption of Safety) status by the European Food Safety Authority (EFSA), exempting them from specific regulatory approval provided they do not possess virulent traits or antibiotic resistance [45,46].

Beyond their antimicrobial function, protective cultures may offer additional technological and functional benefits. These include the production of exopolysaccharides with texturizing properties, reduction of biogenic amines such as histamine, and modulation of gut microbiota in fermented foods containing live cultures [15].

Commercial application of protective cultures in fermented dairy products, such as yogurts, has yielded improvements in microbiological stability, textural maintenance, and shelf life extension, all without compromising physicochemical integrity or sensory characteristics [47].

However, several challenges remain for their broader implementation, notably the standardization of culture composition, stability during storage, compatibility with other starter strains, and performance across varied food matrices. To address these issues, omics technologies (e.g., metagenomics and metabolomics) and ecological modeling approaches are increasingly employed to elucidate microbial interactions and optimize the rational selection and application of protective cultures in complex food systems [14].

### 3.3. Phage Therapy and CRISPR-Based Biocontrol

The use of bacteriophages (phages) and CRISPR-Cas-based systems represents a cutting-edge approach in microbial biocontrol, offering highly specific and environmentally safe alternatives for pathogen management in food systems and processing environments. These strategies embody the principles of precision microbiology, enabling the targeted suppression of undesirable microorganisms without disturbing beneficial microbiota or compromising the sensory quality of foods [48,49].

Bacteriophages are viruses that infect and replicate exclusively within bacteria. Their application in food safety focuses on the selective elimination of pathogenic bacteria such as Listeria monocytogenes, Salmonella enterica, Escherichia coli O157:H7, and Campylobacter jejuni, with their efficacy demonstrated in various products, including ready-to-eat meats, minimally processed vegetables, dairy products, and seafood [42].

Several commercial phage-based products—such as ListShield^TM^, EcoShield^TM^, and PhageGuard—have been approved by regulatory bodies including the U.S. Food and Drug Administration (FDA) and the European Food Safety Authority (EFSA) and are Generally Recognized As Safe (GRAS). These products can be applied via spraying, immersion, or integration into active packaging, without altering the organoleptic properties of foods [48].

Additionally, real-world applications of phage biocontrol are increasingly seen in the food industry. The U.S.-based company *Intralytix* has developed GRAS-approved phage products like *ListShield^TM^* for *Listeria monocytogenes* and *EcoShield^TM^* for *E. coli*, which are applied to ready-to-eat meats and vegetables. Likewise, *Micreos BV* (The Netherlands) manufactures *PhageGuard*, used in the dairy and meat industries to control pathogens like *Listeria* and *Salmonella* without affecting sensory properties

**Figure 1:**
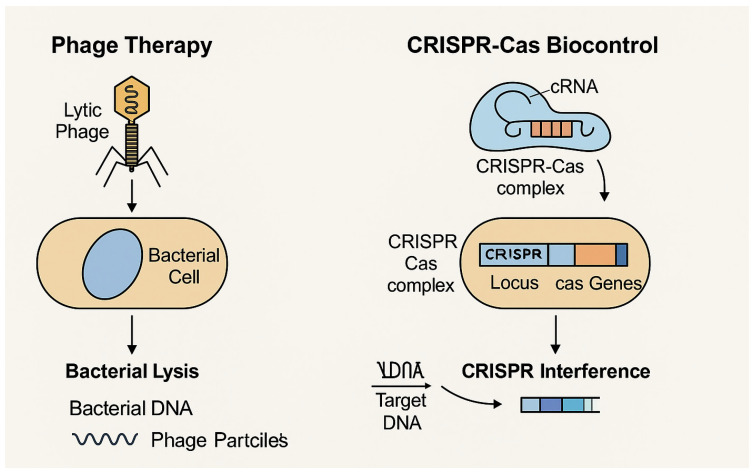
Schematic illustration of biocontrol strategies based on bacteriophages and CRISPR-Cas systems. The mechanisms presented include direct bacterial lysis by lytic phages and gene-targeted disruption via CRISPR interference. Adapted from Sturino and Klaenhammer (2006) and Ghosh et al. (2019) [49,50].

The mechanisms of action of phage therapy and CRISPR-Cas biocontrol are illustrated in Figure 1. This schematic representation is based on the models proposed by Sturino and Klaenhammer (2006) [49], who described engineered bacteriophage resistance systems in bioprocessing, and by Ghosh et al. (2019) [50], who highlighted phages and CRISPR-Cas systems as emerging alternatives to antibiotics.

Key advantages of phage therapy include:

- High specificity, which prevents disruption of non-target microbial communities.
- Self-limiting action, as phages only replicate in the presence of their specific bacterial hosts [51].
- Low risk of cross-resistance with conventional antibiotics.
- Compatibility with mild processing technologies, supporting clean label trends [52].

Nevertheless, some limitations persist. These include the rapid inactivation of phages in complex food matrices, the narrow host range of monovalent phages, and the need for strain-specific regulatory approvals [53]. To overcome these challenges, recent advancements include the development of phage cocktails and genetically engineered phages, which enhance stability, broaden the antibacterial spectrum, and improve the robustness of biocontrol interventions.

CRISPR-Cas systems, originally identified as adaptive immune mechanisms in prokaryotes, have been repurposed into high-precision tools for antimicrobial applications. These systems utilize guide RNAs to direct Cas nucleases to specific genomic sequences, allowing for the selective degradation of bacterial chromosomes, virulence genes, or antibiotic resistance plasmids [54].

Recent studies have demonstrated that CRISPR-Cas systems delivered via engineered bacteriophages can selectively eliminate target pathogens within complex microbial communities—such as those found in fermented foods or ready-to-eat meats—without disturbing the native microbiota [55]. This targeted approach is especially advantageous in products where microbial diversity is integral to sensory quality and functional properties.

Moreover, CRISPR-Cas technology has been employed to silence genes involved in biofilm formation, toxin production, and antimicrobial resistance, reinforcing its potential for use in food safety and industrial sanitation [56].

Despite its promise, the application of CRISPR-Cas in food systems faces regulatory, ethical, and societal challenges. These include concerns about the use of genetically modified organisms (GMOs), traceability, and consumer acceptance. However, the growing demand for precise, low-impact antimicrobial tools is driving translational research in this field, with tangible prospects for industrial implementation in the near future. Table 2 summarizes this information.

## 4. Functional Foods and Health-Promoting Microorganisms

### 4.1. Probiotics, Prebiotics, Synbiotics, and Postbiotics

The growing understanding of the relationship between the gut microbiota and human health has intensified interest in dietary components capable of positively modulating this complex microbial ecosystem. In this context, probiotics, prebiotics, synbiotics, and, more recently, postbiotics have emerged as promising functional tools for health promotion, disease prevention, and innovation in the development of sustainable foods [54,55].

According to the joint definition by the FAO/WHO, probiotics are “live microorganisms which, when administered in adequate amounts, confer a health benefit on the host” [56]. The most studied probiotic strains belong to the genera *Lactobacillus*, *Bifidobacterium*, *Saccharomyces*, *Streptococcus*, and *Lactococcus*, selected based on strict criteria such as resistance to gastric acidity and bile salts, adhesion to intestinal mucosa, immunomodulatory activity, antagonism against pathogens, and proven safety for human consumption [57].

Clinical studies have linked probiotic intake with a range of health benefits, including improvements in gastrointestinal function (e.g., reduction of diarrhea and constipation), modulation of immune responses, influence on the gut-brain axis, and support in the prevention and management of metabolic disorders such as obesity and type 2 diabetes [58]. In the food sector, probiotics have been incorporated into fermented dairy and plant-based products, infant formulas, functional beverages, and nutraceutical supplements, often requiring encapsulation technologies (e.g., microencapsulation or spray drying) to preserve cell viability during processing and storage until consumption.

Prebiotics are defined as “substrates that are selectively utilized by host microorganisms conferring a health benefit”. The most investigated prebiotics include fructooligosaccharides (FOS), galactooligosaccharides (GOS), inulin, and xylooligosaccharides (XOS), as well as plant-derived polyphenols from sources such as grapes, cocoa, and green tea [59]. These compounds preferentially stimulate the growth and metabolic activity of beneficial bacteria—particularly *Bifidobacterium* and *Lactobacillus*—enhancing the production of short-chain fatty acids (SCFAs) such as acetate, propionate, and butyrate, which play essential roles in intestinal homeostasis, immune modulation, and host metabolism.

Synbiotics combine probiotics and prebiotics to synergistically promote the survival and activity of beneficial microbes in the host. They are categorized into *complementary synbiotics*, where the components function independently, and *synergistic synbiotics*, in which the prebiotic selectively enhances the growth and metabolic activity of the co-administered probiotic strain [60]. Synbiotics have demonstrated efficacy in various clinical contexts, including inflammatory bowel disease, antibiotic-associated dysbiosis, and immunonutrition strategies for vulnerable populations such as the elderly and children.

Postbiotics represent a new generation of microbiome-derived ingredients, defined as “preparations of inactivated microorganisms and/or their metabolites that confer health benefits to the host” [61]. These compounds have attracted increasing attention due to their superior stability, safety profile, and compatibility with various food processing conditions when compared to live probiotics [62]. Unlike probiotics, postbiotics do not require viability to be effective, making them ideal for incorporation into heat-treated or shelf-stable food products. Key postbiotic components include short-chain fatty acids (SCFAs), organic acids, microbial cell fragments, peptides, and enzymes, which exhibit immunomodulatory, anti-inflammatory, and antioxidant activities.

Additionally, there is growing scientific consensus on the importance of clearly distinguishing probiotics, prebiotics, and postbiotics in functional food labeling and health claims. According to updated international guidelines, only microbial strains with proven health benefits—supported by human clinical trials and thorough taxonomic identification—should be designated as “probiotics” [63]. This distinction enhances consumer trust and promotes transparency while also supporting regulatory harmonization in the use of microbial ingredients in food systems.

Synbiotics combine probiotics and prebiotics in formulations aimed at improving the survival and metabolic activity of beneficial microorganisms in the host. They are categorized as complementary, where each component acts independently, or synergistic, where the prebiotic is specifically selected to support the probiotic strain [64]. Synbiotics have demonstrated clinical potential in inflammatory bowel disease, antibiotic-induced dysbiosis, and immunonutrition strategies for pediatric and elderly populations [65,66].

Postbiotics, recently defined by ISAPP as “preparations of inanimate microorganisms and/or their components that confer health benefits on the host,” represent a paradigm shift in the use of microbial-based interventions [67]. Unlike probiotics, postbiotics are not dependent on viability for efficacy, offering advantages in terms of safety, stability, and compatibility with industrial food processing [68]. They can be incorporated into heat-treated, shelf-stable, or non-refrigerated formulations while retaining their bioactivity [69].

Unlike probiotics, their functionality does not depend on microbial viability, offering enhanced stability, safety, and technological versatility, especially in food products that undergo thermal treatment or do not require refrigeration.

Key postbiotic components include antimicrobial peptides, organic acids, exopolysaccharides, vitamins, enzymes, and SCFAs, all of which have demonstrated anti-inflammatory, antioxidant, immunomodulatory, and gut barrier-protective effects [70]. Due to their stability and ease of incorporation, postbiotics present regulatory and industrial advantages over live probiotics, making them attractive candidates for the formulation of functional foods with a clean label and proven efficacy.

The future of functional nutrition is expected to move towards precision and personalized formulations, integrating omics technologies (e.g., metagenomics and metabolomics), bioinformatics, and targeted delivery systems. In this context, probiotics, prebiotics, synbiotics, and postbiotics will continue to play a central role in the design of sustainable and science-based food systems tailored to improve public health outcomes.

### 4.2. Technological Challenges and Stability of Functional Microbial Ingredients

The efficacy of probiotics and synbiotics largely depends on their viability and metabolic activity at the time of consumption. This poses significant formulation challenges, especially during food processing, storage, and gastrointestinal transit. Strategies such as microencapsulation, lyophilization, and the use of protective carriers (e.g., resistant starches or lipid-based matrices) are increasingly employed to enhance microbial survival and functional delivery. Moreover, ensuring strain-specific stability without compromising sensory quality remains a priority in the development of functional products [71,72,73,74].

### 4.3. Applications in Personalized Nutrition

Advancements in microbiome research have revealed significant inter-individual variability in microbial composition and metabolic responses to dietary interventions. This has opened the door for personalized nutrition strategies, where probiotics, prebiotics, and synbiotics are tailored to individual gut profiles or health conditions. Tools such as metagenomic sequencing and machine learning are being integrated into clinical practice to guide targeted formulations that optimize host–microbe interactions for specific outcomes [75,76,77,78].

### 4.4. Role in Mental Health and the Gut-Brain Axis

Emerging evidence highlights the influence of gut microbiota on neurological function through the gut-brain axis, implicating microbial metabolites such as SCFAs, neurotransmitter precursors, and immune mediators. Certain probiotic strains—sometimes referred to as “psychobiotics”—have shown promise in modulating mood, reducing anxiety, and improving cognitive performance. These findings suggest potential for probiotic and postbiotic interventions in mental health management, although further clinical validation is needed [79,80,81].

### 4.5. Environmental and Sustainability Perspectives

Incorporating microbiome-based ingredients into food systems also contributes to sustainability goals. Fermentation processes using probiotic or postbiotic-producing microbes can valorize agricultural by-products, reduce food waste, and lower the environmental footprint of production. Additionally, the development of self-stable, non-refrigerated postbiotic formulations aligns with the reduction of energy demands across the food supply chain [82,83,84,85]. Table 3 summarizes this content.

## 5. Regulatory and Technological Advances in Microbial Ingredients for Food Innovation

The advancement of microbial innovations applied to food—including functional fermented foods, protective cultures, biocontrol, and precision fermentation—requires not only technological development but also alignment with specific regulatory frameworks, overcoming technical barriers and understanding the expectations of modern consumers. The consolidation of these products in the market depends on the interaction between science, regulation, and social acceptance [86,87,88].

In the European Union, the authorization of microorganisms or their derivatives for use in food follows the criteria established by the EFSA, through the QPS (Qualified Presumption of Safety) status. This system evaluates toxicological safety, usage history, and the absence of virulence and antimicrobial resistance genes [38]. Products such as probiotics, microbial enzymes, and bacteriocins can also be classified as novel foods, requiring specific evaluation before commercialization [89,90,91].

In the United States, the FDA system classifies substances and microorganisms as GRAS (Generally Recognized As Safe). Companies can submit safety assessment dossiers or use strains with a proven history of use. Postbiotic ingredients and bioprotective cultures have been classified based on scientific data and traditional usage precedents [92,93].

In Brazil, ANVISA regulates probiotics and functional ingredients through Normative Instruction No. 60/2020, requiring scientific evidence of efficacy and safety, as well as the standardization of strains and dosages. Labeling and health claims must follow the guidelines of RDC No. 429/2020, promoting transparency and traceability [18,94].

Despite regulatory advances, there are still gaps in regulations for ingredients such as postbiotics, proteins produced by precision fermentation, phages, and CRISPR systems, which incorporation depends on case-by-case evaluations and updated legal frameworks [21,95].

The transition of fermentation processes from the laboratory to industrial scale presents challenges such as the selection and maintenance of stable strains, reproducible control of fermentation conditions, ensuring cell viability (in the case of probiotics), and optimizing the yield of target metabolites [67].

Technologies such as automated bioreactors, kinetic modeling, genetic editing of industrial strains, and spray drying or fluidized bed encapsulation have been incorporated to improve the efficiency, cost-effectiveness, and functional stability of products with live or inactivated microorganisms [18].

Furthermore, the application of omics tools (metagenomics, proteomics, and metabolomics) associated with artificial intelligence has enabled the customization of ingredients based on the target microbiome profile, opening up opportunities for functional foods with individualized applications and greater clinical efficacy [15,95,96].

The global market for microbial functional foods has grown exponentially, driven by consumers seeking natural products with health claims, clean labeling, and lower environmental impact. It is estimated that the probiotics and synbiotics sector will generate over USD 80 billion by 2026, with a focus on yogurts, plant-based beverages, snacks, and encapsulated supplements [97].

Behavioral studies indicate that consumers value attributes such as naturalness of ingredients, local or artisanal origin, sustainability in production, and transparency in functional claims [58].

However, the acceptance of emerging technologies, such as precision fermentation, synthetic postbiotics, and foodborne phages, still faces resistance from more conservative consumers, requiring communication strategies based on scientific education, trust in certifications, and regulatory transparency [98].

## 6. Evidence-Based Insights into Microbial Innovations

Food microbiology stands at the forefront of some of the most promising strategies for building sustainable, resilient, and functional food systems. Throughout this review, we have examined evidence highlighting the transformative potential of microorganisms in food production, preservation, and functionalization, bringing together technological innovation, public health goals, and environmental responsibility [83,84,99].

Microbial fermentation, whether in its traditional forms or through precision approaches, has shown remarkable versatility and low environmental impact in converting plant-based substrates and agro-industrial by-products into safe, nutritious, and value-added foods. The use of starter cultures, synergistic microbial consortia, and metabolic engineering enables process control, standardization, and functional customization across different production scales and cultural contexts [90,100,101].

Microbial biocontrol, through mechanisms such as competitive exclusion, bacteriocins, bacteriophages, and CRISPR-Cas-based technologies, offers a promising alternative to chemical preservatives. These strategies provide natural, targeted solutions for enhancing food safety and shelf life, especially in minimally processed products, and are generally well accepted by consumers seeking clean label alternatives [97,98,99,100,101,102].

In the field of functional foods, the integration of probiotics, prebiotics, synbiotics, and postbiotics has been associated with improvements in gut microbiota composition and a range of health indicators, including metabolic, immune, and cognitive functions. Developing such products with sustainability in mind—using local raw materials, intelligent encapsulation methods, and waste valorization—reinforces their relevance as essential components of a healthier, more ethical, and adaptable diet for the 21st century [83,90,100,101,102,103,104,105,106,107].

Despite their potential, the widespread adoption of these microbial innovations still faces key challenges, including methodological harmonization, clinical validation of health benefits, regulatory alignment, and consumer education. The integrated application of omics technologies, artificial intelligence, and environmental bioprospecting could accelerate discoveries; support personalized interventions; and broaden the ethical, safe, and scalable application of microbial solutions [108,109,110].

**Figure 2:**
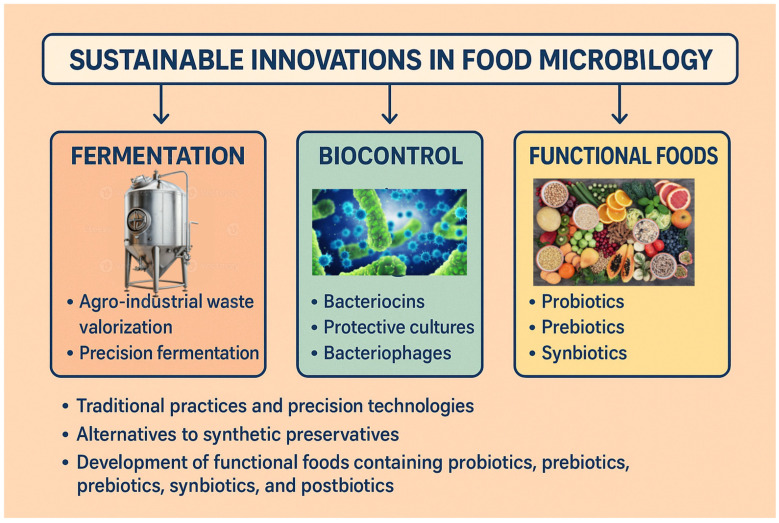
Sustainable innovations in food microbiology.

In conclusion, food microbiology—when grounded in sustainability and powered by emerging technologies—has the capacity to drive the transition toward a new food paradigm that is healthier, regenerative, and evidence-based. Achieving this vision will require interdisciplinary collaboration among researchers, regulatory bodies, industry, and society, as well as sustained investment in applied science, open innovation, and public policies that promote the bioeconomy and global food security (Figure 2).

## 7. Future Trends and Research Gaps

Food microbiology, increasingly aligned with sustainability and precision health, is entering a transformative phase driven by the convergence of advanced biotechnological platforms; unconventional microbial resources; and the pressing need for scientific, technological, and regulatory standardization. This multifaceted evolution is pivotal for translating laboratory discoveries into robust, safe, and scalable innovations with real-world applications across the food sector [111,112,113,114].

The rapid development and integration of omics technologies—including metagenomics, transcriptomics, proteomics, and metabolomics—have profoundly reshaped our understanding of microbial ecosystems in food matrices. These tools allow for high-resolution analyses of the composition, functionality, and ecological interactions of microbial consortia. Advanced techniques such as next-generation sequencing (NGS) and high-resolution mass spectrometry are now routinely employed to characterize microbial communities in fermented foods, detect emerging spoilage or pathogenic agents, and identify novel bioactive compounds or resistance mechanisms [115,116,117].

Simultaneously, the integration of artificial intelligence (AI) and machine learning (ML) into microbiome research has opened new frontiers in predictive modeling, enabling the simulation of microbial interactions, metabolic pathway optimization, and customization of functional foods in accordance with individual microbiome profiles [118,119,120]. Computational platforms such as QIIME, PICRUSt, and GNPS are being increasingly linked with expanding genomic and metabolomic databases, enhancing the accuracy of metabolic function prediction and enabling the design of microbial consortia with targeted health applications [74,121,122,123,124].

These multidisciplinary strategies are driving the development of a new generation of precision-functional foods—tailored formulations incorporating microbial strains or metabolites aimed at specific physiological goals such as glycemic regulation, modulation of inflammation, immune enhancement, or neurological well-being [67,68,69,70,71,72,73,74,75,76,77,78,79,80,81,82,83,84,85,86,87,88,89,90,91,92,93,94,95,96,97,98,99,100,101,102,103,104,105,106,107,108,109,110,111,112,113,114,115,116,117,118,119,120,121,122,123,124,125,126].

In parallel, extreme environments, including hydrothermal vents, hypersaline lakes, polar ice caps, marine sediments, and arid ecosystems, are being increasingly explored as reservoirs of novel microbial biodiversity. Microorganisms adapted to extreme conditions (e.g., halotolerant, thermophilic, or psychrophilic strains) possess unique enzymatic systems and bioactive metabolites, many of which exhibit antimicrobial, antioxidant, or biopreservative activities with potential applications in food preservation and functionalization [127,128,129,130,131].

Tropical regions, especially biodiversity hotspots such as the Brazilian Atlantic Forest, Amazon Basin, and Cerrado biome, represent strategic frontiers for the bioprospecting of autochthonous probiotics, new bacteriocins, and biocontrol agents adapted to local climatic conditions and traditional food substrates [132,133,134]. However, the ethical and sustainable exploration of these microbial resources must strictly adhere to international frameworks, particularly the Nagoya Protocol and national legislation governing access to genetic heritage and benefit-sharing [135,136,137,138,139,140,141,142].

Despite notable advances, there are still considerable research and regulatory gaps that hinder the consolidation of microbiome-derived innovations in food systems. One major limitation is the lack of methodological standardization in the isolation, characterization, and application of microbial strains and consortia. This heterogeneity impedes cross-study comparisons, reduces reproducibility, and poses significant barriers to clinical validation and regulatory approval [15,62].

There is an urgent need for internationally harmonized guidelines encompassing key parameters such as accurate taxonomic identification and strain-level quantification, determination of cell viability and bioactivity in vivo, design and execution of robust randomized clinical trials (RCTs), evaluation of long-term stability during processing and storage, and comprehensive toxicological and genomic safety assessments [137,138].

Furthermore, while the therapeutic potential of postbiotics, personalized synbiotics, and next-generation fermented foods has been increasingly documented, the lack of long-term studies and high-quality clinical trials—especially in vulnerable populations such as elderly individuals, children, and immunocompromised patients—continues to limit their clinical translation and acceptance by regulatory authorities [31,81,142,143].

Bridging these gaps requires the development of integrated translational research platforms that connect basic science, industrial development, and regulatory science. These platforms should promote multi-stakeholder collaboration, ensure equitable benefit-sharing, and support the democratization of sustainable microbial solutions in food systems. Ultimately, aligning microbiome innovation with public health priorities, consumer values, and planetary boundaries will be key to unlocking its full potential in shaping the future of food [81,108,144,145].

## 8. Conclusions

The integration of traditional fermented foods with innovative microbial biocontrol strategies provides a robust and sustainable foundation for advancing food quality, safety, and functionality. Fermentation processes, driven by diverse microbial consortia, not only preserve food but also enhance its nutritional and functional properties, contributing to both human health and cultural continuity. At the same time, emerging precision biocontrol tools—such as protective cultures, bacteriophages, and CRISPR-Cas systems—represent a clear evolution from conventional preservation techniques toward a more targeted and adaptive microbiology.

These innovations align with clean label trends, evolving regulatory frameworks, and the urgent need for resilient food systems in the face of global challenges. Nevertheless, their widespread implementation requires further investigation into strain specificity, process integration, consumer acceptance, and policy alignment. By bridging traditional ecological knowledge with advanced molecular technologies, the food sector is poised to deliver safer, more effective, and personalized solutions. Interdisciplinary research will not only optimize these tools across diverse food matrices but will also shape the future of sustainable and functional food innovation.

## Figures and Tables

**Table 1:** Overview of representative traditional fermented foods, their microbial ecology, fermentation type, origin, and key metabolic products.

Fermented Product	Main Microorganisms	Fermentation Type	Country of Origin	Key Metabolic Products
Kefir	*Lactobacillus* spp., *Kluyveromyces marxianus*	Spontaneous	Caucasus region	Organic acids, ethanol, CO_2_
Tempeh	*Rhizopus oligosporus*	Controlled	Indonesia	Bioactive peptides, hydrolytic enzymes
Miso	*Aspergillus oryzae*, *Zygosaccharomyces rouxii*	Controlled	Japan	Free amino acids, vitamins, aromatic compounds
Kimchi	*Lactobacillus* spp., *Leuconostoc* spp.	Spontaneous	Korea	Organic acids, phenolic compounds
Sauerkraut	*Leuconostoc mesenteroides*, *Lactiplantibacillus plantarum*	Spontaneous	Germany	Lactic acid, bacteriocins

**Table 2:** Applications of protective lactic acid bacteria (LAB) in food matrices, target pathogens, and inhibitory mechanisms. Data compiled by the authors based on sources [36,38,39,40,41,42,43,44,45,46,47,48,57].

Strategy/Technology	Main Mechanisms	Microorganisms or Agents Involved	Food Applications	Advantages	Challenges/Limitations
Section 3.1. Natural Antimicrobials Produced by Microorganisms	Bacteriocins, organic acids, hydrogen peroxide, biotransformation of phenolics	Lactic Acid Bacteria (LAB), e.g., *Lactococcus lactis*, *L. plantarum*, *P. acidilactici*	Cheeses, processed meats, ready-to-eat vegetables, refrigerated juices	Natural alternative to synthetic preservatives, “clean label”, microbial safety	Thermal instability, limited spectrum of activity, variable efficacy across food matrices
Section 3.2. Competitive Exclusion and Protective Cultures	Nutrient and niche competition, production of antimicrobial metabolites	*L. plantarum*, *L. rhamnosus*, *P. acidilactici*, *Leuconostoc mesenteroides*, *L. sakei*, *C. maltaromaticum*	Fresh cheeses, fermented meats, refrigerated dairy products, minimally processed vegetables	Antibiotic-free, safe (GRAS/QPS), preservation of sensory characteristics	Need for standardization, compatibility with starter cultures, stability during storage
Section 3.3. Phage Therapy and CRISPR-based Biocontrol	Targeted bacterial lysis, gene editing of virulence/resistance factors	Bacteriophages (e.g., ListShield^TM^, EcoShield^TM^), CRISPR-Cas systems (via engineered phages)	Ready-to-eat meats, fresh vegetables, dairy products, seafood	High specificity, preserves beneficial microbiota, no sensory alteration	Narrow host range, inactivation in complex food matrices, regulatory and consumer acceptance barriers

**Table 3:** Overview of the functional food components, their mechanisms of action, and health-related applications. Data compiled by the authors based on Verma et al. (2022) [84], Abedin et al. (2024) [85], and additional literature sources [66,67,68,69,70,71,72,73,74,75,76].

Category	Definition	Key Components	Mechanisms of Action	Applications	Challenges/Advantages
Probiotics	Live microorganisms that, when administered in adequate amounts, confer a health benefit on the host.	*Lactobacillus*, *Bifidobacterium*, *Saccharomyces*, *Streptococcus*, *Lactococcus*	Modulate gut microbiota, improve barrier function, stimulate immune system, inhibit pathogens	GI health, metabolic disorders, immune modulation, brain-gut axis, functional foods	Require viability; need encapsulation for stability during processing and storage
Prebiotics	Substrates selectively utilized by host microorganisms conferring health benefits.	FOS, GOS, inulin, XOS, polyphenols from grapes, cocoa, green tea	Promote growth of beneficial bacteria; increase SCFA production (acetate, propionate, butyrate)	Gut health, mineral absorption, modulation of inflammation	Selectivity of fermentation; often fiber-based ingredients; stable during processing
Synbiotics	Combination of probiotics and prebiotics that beneficially affect the host by improving microbial survival/activity	Complementary (independent action) or synergistic (prebiotic enhances co-administered probiotic)	Enhance colonization and metabolic activity of beneficial microbes	IBD, dysbiosis, nutrition for elderly and children, clinical and dietary interventions	Must ensure compatibility between strains and substrates; growing personalized formulations
Postbiotics	Preparations of inactivated microorganisms and/or their metabolites that confer health benefits to the host.	SCFAs, organic acids, antimicrobial peptides, vitamins, exopolysaccharides, enzymes	Provide anti-inflammatory, antioxidant, immunomodulatory, and barrier-protective effects without requiring viability	Stable functional foods, clinical nutrition, safer alternatives to live probiotics	High stability; suitable for thermally processed foods; reduced regulatory concerns compared to live microbes

## Data Availability

No new data were created or analyzed in this study.
